# Structure matters: commensal *Phocaeicola vulgatus* lipopolysaccharide induces attenuated microglial activation and preserves neuronal integrity

**DOI:** 10.3389/fncel.2026.1796397

**Published:** 2026-04-14

**Authors:** Valentina Mazziotti, Luca De Simone Carone, Francesca Olmeo, Fabrizio Chiodo, Alba Silipo, Antonio Molinaro, Flaviana Di Lorenzo

**Affiliations:** 1 Department of Chemical Sciences, University of Naples Federico II, Naples, Italy; 2 CEINGE-Biotecnologie Avanzate Franco Salvatore, Naples, Italy; 3 Bio-Organic Chemistry Unit, Institute of Biomolecular Chemistry CNR, Naples, Italy; 4 Department of Molecular Cell Biology and Immunology, Amsterdam UMC, Vrije Universiteit Amsterdam, Amsterdam, Netherlands

**Keywords:** gut-brain axis, lipopolysaccharide (LPS), Microglia, neuroinflammation, Phocaeicola vulgatus

## Abstract

Lipopolysaccharides (LPSs) from Gram-negative bacteria are widely used to model neuroinflammation *in vitro* and *in vivo*. However, this paradigm assumes that all LPS chemotypes are uniformly pro-inflammatory, despite significant structural diversity between enterobacterial pathogens and gut-resident commensals. Whether microglia can discriminate among these chemotypes remains largely unexplored. We performed a comparative analysis of canonical *Escherichia coli* LPS and commensal-derived *Phocaeicola vulgatus* LPS in murine (BV2) and human (HMC3) microglial cells. Pro-inflammatory mediators were quantified by ELISA, and TLR4-downstream signaling was assessed by western blotting. Conditioned media (CM) from LPS-treated BV2 and HMC3 cells was applied to PC12 neuronal cells to evaluate cell viability and differentiation by immunofluorescence. In BV2 microglial cells, *P. vulgatus* LPS did not induce nitric oxide (NO) production or iNOS expression. In both BV2 and HMC3 cells, it failed to trigger pro-inflammatory cytokine release or TLR4 pathway activation. CM from *E. coli*-treated microglia disrupted MAP2 expression in PC12 neurons, whereas media from *P. vulgatus*-treated microglia did not. Overall, our data argue that “LPS-induced neuroinflammation” is not a universal phenomenon, but a chemistry-dependent outcome shaped by specific LPS structures. This study therefore highlights the need to consider LPS structural diversity in neuroinflammation models, particularly in the context of gut-brain communication.

## Introduction

Microglia are the resident immune cells of the central nervous system (CNS) and act as primary sentinels of inflammatory and infectious cues (Dadwal and Heneka, 2024). Through the engagement of pattern-recognition receptors, among which Toll-like receptor 4 (TLR4), microglia rapidly translate extracellular danger signals into intracellular inflammatory programs that significantly influence neuronal function and survival (Doens and Fernández, 2014; Gao et al., 2023; Acioglu and Elkabes, 2025). For this reason, lipopolysaccharides (LPSs) from Gram-negative bacteria, the prototypical ligands of TLR4, have become the most widely used experimental tools to model microglial activation and neuroinflammation *in vitro* and *in vivo* (Batista et al., 2019; Brown, 2019; Brown and Heneka, 2024). Exposure to canonical enterobacterial LPSs, typically derived from *Escherichia coli* or *Salmonella*, robustly activates TLR4-dependent signaling cascades, including NF-κB, MAPK, in particular the Erk 1/2 branch, and STAT pathways, leading to nitric oxide (NO) production and the release of pro-inflammatory cytokines (Lu et al., 2008; Di Lorenzo et al., 2022; Page et al., 2022). This experimental paradigm has been instrumental in dissecting inflammation-driven neural dysfunction, as reflected by the extensive literature explicitly referring to “LPS-induced neuroinflammation” (Singh et al., 2022; Skrzypczak-Wiercioch and Sałat, 2022; da Silva et al., 2024). However, it is based on an implicit and rarely questioned assumption: that LPSs represent a uniform and intrinsically pro-inflammatory stimulus for microglia. In reality, LPSs are structurally diverse molecules whose immunological activity is shaped by subtle variations in their chemical architecture (Di Lorenzo et al., 2015; Di Lorenzo et al., 2022). Nevertheless, most neuroinflammation studies employ a narrow set of highly immunostimulatory enterobacterial LPS chemotypes, with a well-defined chemistry, which may fail to capture the diversity of microbial signals encountered by the host under physiological conditions (Vargas-Caraveo et al., 2020; Singh et al., 2022; Skrzypczak-Wiercioch and Sałat, 2022; da Silva et al., 2024).

Recent advances in microbiome research have highlighted the profound influence that gut microbes and their metabolites exert on brain function, giving rise to the concept of the gut-brain axis, i.e., a bidirectional communication network linking the gastrointestinal tract and the CNS (Ma et al., 2019; Suganya and Koo, 2020; Morais et al., 2021; Keane et al., 2025). In this context, the relevance of LPS diversity becomes particularly evident, as host exposure to microbial products derives predominantly from gut-resident commensal bacteria (i.e., the gut microbiota) rather than overt pathogens. These commensals can act as potent regulators of glial functions and immune tone within the CNS (Loh et al., 2024; Keane et al., 2025). Strikingly, increasing evidence indicates that LPSs from gut microbiota members challenge the long-standing paradigm of these molecules as uniformly pro-inflammatory signals, as they often exhibit distinctive chemical structures compared to classical enterobacterial, and typically pathogenic, LPSs. These differences include reduced acylation and phosphorylation states, as well as atypical sugar epitopes, which have been linked to attenuated TLR4 activation, biased signaling responses, and, in some cases, active immunomodulatory effects (Di Lorenzo et al., 2019; Di Lorenzo et al., 2020; Shimoyama et al., 2021; Di Lorenzo et al., 2022; Pither et al., 2022; Pither et al., 2024; De Chiara et al., 2025; De Simone Carone et al., 2025; Nieto-Fabregat et al., 2025; Tiemblo Martín et al., 2025; Tiemblo-Martin et al., 2025). Moreover, in peripheral immune cells, several microbiota-derived LPSs have been shown not only to weakly engage the TLR4/MD-2 complex, but also to interact with additional receptors, including TLR2 and carbohydrate-binding proteins such as lectins, enabling receptor crosstalk and fine-tuning of inflammatory responses in a strictly structure-dependent manner (Di Lorenzo et al., 2020; Di Lorenzo et al., 2022; Pither et al., 2022; Pither et al., 2024; De Chiara et al., 2025; Nieto-Fabregat et al., 2025; Rai et al., 2025; Tiemblo Martín et al., 2025; Tiemblo-Martin et al., 2025). Together, these insights are reshaping our conceptual understanding of LPS biology, highlighting how LPS chemistry can encode immunological functions far beyond inflammation induction. This also opens a largely unexplored frontier in neuroscience research, where LPS-based models remain widely used but rarely interrogated for structural diversity.

In this context, *Phocaeicola vulgatus* (formerly *Bacteroides vulgatus*), a prevalent member of the human gut microbiota, represents a particularly compelling case study. Its LPS is characterized by a hypoacylated and monophosphorylated lipid A portion, structural traits known to dramatically dampen canonical TLR4 signaling, together with glycan epitopes that contribute to its potent immunomodulatory profile (Di Lorenzo et al., 2020; Nieto-Fabregat et al., 2024; Rai et al., 2025). Consistent with these features, *P. vulgatus* LPS has been shown to elicit minimal pro-inflammatory cytokine production in human dendritic cells while promoting robust IL-10 release, a hallmark of tolerogenic immune engagement (Di Lorenzo et al., 2020). Even more interestingly, *in vivo*, this commensal-derived LPS alleviates intestinal inflammation in murine models of colitis by inducing a specialized form of LPS tolerance mediated through the MD-2/TLR4 axis in lamina propria CD11c^+^ cells (Steimle et al., 2019). Despite these emerging insights, whether structurally distinct commensal LPS chemotypes are sensed by microglia as inflammatory stimuli, or instead elicit qualitatively different activation programs, remains largely unexplored.

Here, we report for the first time a direct side-by-side comparison of enterobacterial and commensal-derived LPS in murine and human microglial *in vitro* systems. In addition, we evaluated the effects of conditioned media from LPS stimulated microglia on PC12 neuronal cells, aimed at exploring the impact of *P. vulgatus* LPS on neuronal viability and differentiation. By integrating analyses of inflammatory mediators and key TLR4-associated signaling pathways, we demonstrate that *P. vulgatus* LPS fails to elicit a canonical pro-inflammatory microglial activation program. Consistently, *P. vulgatus* LPS did not impair neuronal growth or homeostasis. These observations provide direct evidence that microglial inflammatory responses are not an intrinsic property of LPSs *per se*, but critically depend on their chemistry, challenging the widespread assumption that all LPSs act as pro-inflammatory drivers in neuroinflammation models.

## Methods

### Reagents and cell lines

*P. vulgatus* LPS was obtained as previously described (Di Lorenzo et al., 2020; Pither et al., 2023). Briefly, LPS was extracted from lyophilized bacterial cells using the hot phenol-water method, followed by enzymatic digestion with DNase, RNase, and proteases to remove residual contaminants (Di Lorenzo et al., 2020; Pither et al., 2023). The extract was further purified by size-exclusion chromatography and ultracentrifugation. Additional purification steps were performed, as previously reported for this LPS (Di Lorenzo et al., 2020), to remove phospholipids and potential lipoprotein contaminants that could activate other receptors, such as TLR2. The murine BV2 microglial cell line (bladder carcinoma cell line-derived, viral oncogene-transformed; #305156) and the human microglial clone-3 cell line (HMC3; #300102) were purchased from Cytion Biosciences (Heidelberg, Germany). The rat pheochromocytoma cell line (PC12; #910001) was purchased from CH3 BioSystems (United States) and distributed in Europe by Tebu-bio (Le Perray-en-Yvelines, France). BV2 and HMC3 were maintained in Dulbecco’s Modified Eagle’s Medium (DMEM), high glucose, with pyruvate (Gibco, Grand Island, NY, United States, #11995073), supplemented with 10% heat inactivated fetal bovine serum (FBS; Gibco, #10270-106), 2 mM L-glutamine (Gibco, #25030-024), and 1% penicillin/streptomycin (Pen/Strep; Gibco, #15140-122). PC12 were cultured in Roswell Memorial Park Institute-1640 (RPMI-1640) medium (Gibco, #11875093), 10% Horse Serum (EuroClone, #ECS0090L); 5% FBS. For neuronal differentiation, the medium was replaced with RPMI containing 1% horse serum and 50 ng/mL nerve growth factor (NGF; R&D Systems, #556-NG). Prior to seeding, culture surfaces were coated with laminin (Corning^®^, 1 mg, #354232) to support cell attachment. All cell lines were maintained in humidified 5% CO_2_/95% air environment at 37°C.

### Cell viability assays

Cell viability was assessed using a 3-(4,5-dimethylthiazol-2-yl)-2,5-diphenyltetrazolium bromide (MTT) assay. Cells were seeded in 96-well plates at 1 × 10^4^ cells/well and treated for 24 h with *E. coli* LPS (LPS-EB ultrapure; InvivoGen) or *P. vulgatus* LPS at concentrations of 0.1, 1, 10, or 100 ng/mL. After treatment, the medium was removed, and the cells were incubated with MTT reagent (Thermo Fisher Scientific, Waltham, MA, United States, 0.25 mg/mL) for 3 h at 37 °C. Formazan crystals in the cells were dissolved with dimethyl sulfoxide (DMSO), and absorbance was measured at 490 nm using TECAN Infinite M Plex spectrophotometer (Tecan, Grödig, Austria).

### Nitrite assay

Accumulation of nitrite (NO_2_^–^), an indicator of NO production, in BV2 supernatants was evaluated by the Griess reaction (Zhao et al., 2016). Cells were seeded in 96-well plates at 1 × 10^4^ cells per well and treated with different concentrations of *E. coli* LPS or *P. vulgatus* LPS for 24 h. Supernatants (100 μL) were mixed with 100 μL Griess reagent (Thermo Fisher Scientific, #328670500) and incubated at room temperature for 10 min. Absorbance was read at 540 nm using a TECAN Infinite M Plex spectrophotometer (Tecan, Grödig, Austria). Nitrite concentrations were calculated from a sodium nitrite standard curve (Sigma-Aldrich, #S2252) and expressed in micromolar.

### Cytokines quantification

Cytokine levels (pg/mL) in BV2 and HMC3 supernatants were measured following *E. coli* or *P. vulgatus* LPS stimulation using the ultrasensitive ELLA-Simple Plex platform (Protein Simple, Bio-Techne, San José, CA, United States), with a commercially available microfluidic cartridge for mouse (ST01C-MP-004435) and for human (ST01A-PS-003229) IL-1β, IL-6, and TNF-α detection. IL-10 was quantified using DuoSet ELISA Kit (R&D systems, Abingdon, United Kingdom) for mouse (#DY417) and for human (#DY217B) for BV2 and HMC3 supernatants, respectively.

### Western blot

For BV2 and HMC3 cells, 50,000 cells were seeded per well in a 24-well plate. For the differentiation experiments, 20,000 cells were seeded and lysed after 14 days in differentiation medium. RIPA buffer (50 mM Tris-HCl pH 7.4, 150 mM NaCl, 1% NP-40, 0.5% sodium deoxycholate, 0.1% SDS, 2 mM EDTA and 100X protease inhibitors; Sigma-Aldrich) was applied to homogenize and lyse treated cells. Lysates were incubated on ice for 20 min, centrifuged at 14,000 rpm for 15 min at 4°C, and total proteins in supernatants were quantified using the Pierce™ BCA protein assay kit (ThermoScientific, #23227). Equal amounts of protein were electrophoretically separated using 7.5% polyacrylamide gels and transferred to polyvinylidene fluoride (PVDF) membrane (Bio-Rad, Hercules, CA, United States, #1620177). Membranes were blocked with 5% non-fat dry milk, and then incubated overnight at 4°C with the following primary antibodies (Cell Signaling Technology, Danvers, MA, United States): rabbit anti-MAP2 (#8707; 1:1,000), rabbit anti-iNOS (#nb300-605), rabbit anti-STAT3 (#12640, 1:1,000), rabbit anti-p-STAT3 (#32143, 1:1,000), rabbit anti-Nf-kB p65 (#8242, 1:500), rabbit anti-p-Nf-kB p65 (#3033, 1:500), rabbit anti-Erk1/2 (#4695, 1:1,000), rabbit anti-p-Erk1/2 (#9101, 1:1,000), rabbit p-IkB α Ser32 (#2859, 1:1,000). Mouse anti-GAPDH (ThermoFisher Scientific, #MA5-15738, 1:3,000) or α-tubulin (Cell Signaling, #3873, 1:3,000) were used as housekeeping proteins to normalize total protein levels. Subsequently, after washing with TBS-Tween 0.1% (10 min/time) membranes were incubated 1 h at room temperature with horseradish peroxidase (HRP)-conjugated secondary antibodies (Merck/Sigma Aldrich, Rahway, NJ, United States). The immunoreactive bands were visualized by enhanced chemiluminescence (ECL) substrate (GE Healthcare Amersham™, Amersham, United Kingdom, RPN2106) by using the Azure 600 Imaging System (Azure Biosystems, Inc., United States).

### Immunofluorescence

Microtubule-Associated Protein 2 (MAP2), a dendritic neuronal marker, and Ionized calcium-Binding Adapter molecule-1 (Iba-1), a canonical microglial marker, were used to identify neuronal PC12 cells and microglial BV2/HMC3 cells, respectively. For immunofluorescence analysis, cells were seeded at the same density used for the Western blot experiments. Cells were washed once with 1X Phosphate-Buffered Saline (PBS) and fixed in 4% paraformaldehyde (PFA) for 15 min at RT. After fixation, cells were washed three times with 1X PBS and permeabilized with 1X PBS/0.1% Triton X-100 (PBST) for 10 min at room temperature (RT). Then, cells were saturated with a blocking solution containing 5% FBS + PBST for 45 min at RT and then incubated for 1 h at RT with the following primary antibodies diluted in blocking solution: rabbit anti-MAP2 (Cell Signaling Technology, #8707, #1:500) for PC12 cells, rabbit anti-Iba1 (#17198, 1:500) for BV2 and HMC3 cells. After three washes with 1X PBS, cells were incubated 1 h at RT with secondary antibodies AlexaFluor 488 and AlexaFluor 594 (Life Technologies, Carlsbad, CA, United States, 1:500). For nuclear staining, the cells were incubated with DAPI (Thermo Fisher Scientific, #62248, 1:1,000) for 5 min. The slide was finally washed, and the cells were mounted with SlowFade Gold Antifade reagent (Thermo Fisher Scientific, #S36937) for microscopic examination. Fluorescence images were acquired using a OL3000TFL biological microscope (Zetalab, Italy). For the quantification of MAP2^+^ PC12 neurons and Iba1^+^ BV2/HMC3, cell count values are expressed as percentage relative to the NS condition. A semi-automated densitometric analysis was performed on Iba-1^+^ cells to calculate the mean fluorescence intensity normalized to the positive signal area. Immunofluorescence images were analyzed using ImageJ software (version 1.53t, National Institutes of Health, Bethesda, MD, United States.^1^ For negative controls, samples were incubated with secondary antibody only (Supplementary Figure 1).

### LPS coated ELISA assay

Human plasma was taken during the isolation of human peripheral blood mononuclear cells (PBMCs) from buffy coats of healthy donors in accordance with the Declaration of Helsinki (Centro Trasfusionale P.O. San Paolo, Napoli). ELISA nunc MaxiSorp™ 96 well plates (Thermo Fisher Scientific, #10565981) were coated with LPS from *E. coli* and *P. vulgatus* at a final concentration of 10 μg/mL in 1X PBS. A total volume of 100 μL per well was added and plates were incubated overnight at 4 °C. The following day, wells were washed with PBS or PBS-T and subsequently blocked with blocking solution (0.5% BSA in PBS) for 1 h at room temperature. Plasma samples from healthy donors (*n* = 4), diluted 1:500 in blocking solution (100 μL per well), were then added and incubated for 1 h. After washing, anti-human IgG HRP conjugated secondary antibodies were applied (Thermo Fisher Scientific, #31410, 1:2,000). Absorbance was read at 450 nm using a TECAN Infinite M Plex spectrophotometer.

### Statistical analysis

All data are expressed as mean ± SEM and represent at least three independent experiments. Statistical significance between LPS treatments were assessed using one-way ANOVA for multiple comparisons. *Post hoc* analyses were performed with Tukey’s multiple comparison test. Analyses were conducted using Prism software version 10.1.1(270) (GraphPad, San Diego, CA, United States). *P* < 0.05 was considered statistically significant.

## Results

### Commensal *P. vulgatus* LPS fails to trigger classical NO-iNOS inflammatory responses in BV2 microglia, in contrast to enterobacterial LPS

**FIGURE 1:**
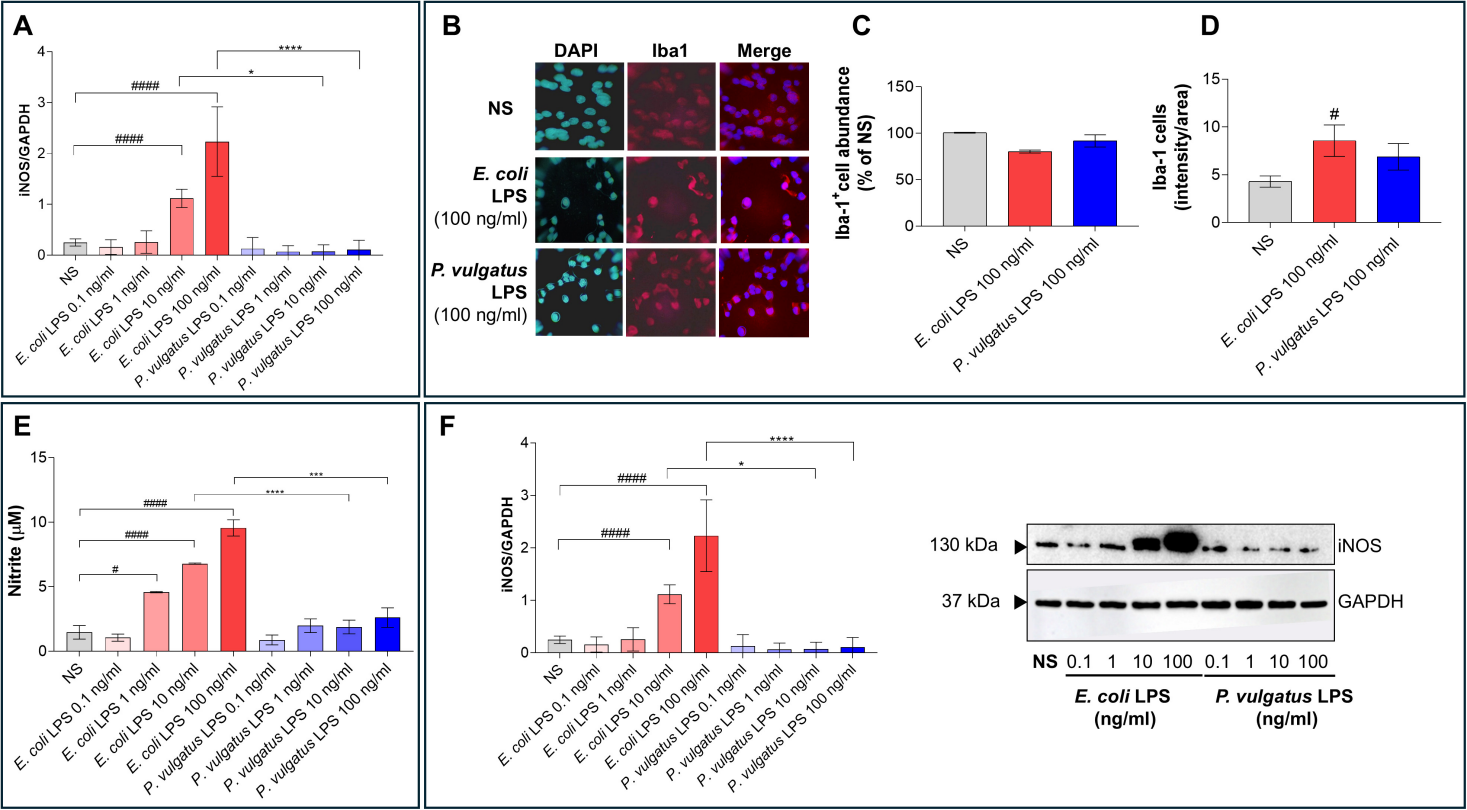
Differential effects of *E. coli* and *P. vulgatus* LPS on BV2 microglial cells. **(A)** BV2 cell viability after stimulation with increasing concentrations of *E. coli* or *P. vulgatus* LPS (0.1–100 ng/mL) using the MTT assay. Unstimulated cells (NS) served as negative control. **(B)** Immunofluorescence analysis of microglial marker Iba-1 (red) to evaluate cell morphology and number following exposure to 100 ng/mL of LPS (scale bar = 100 μm). **(C)** The relative number of Iba 1^+^ cells from the experiments in **(B)**, normalized to NS (set at 100%). **(D)** Iba-1 immunofluorescence intensity was calculated as the mean area of the positive signal per cell. **(E)** Nitrite quantification and **(F)** western blot analysis of iNOS expression. Data are presented as mean ± SEM. Statistical significance was assessed by ordinary one-way ANOVA followed by Tukey’s multiple-comparison test. ^#^*p* < 0.05; ^####^*p* < 0.0001 vs. NS; *p < 0.05; ***p < 0.001, ****p < 0.0001 vs. LPS.

We initially evaluated potential cytotoxic effects by exposing BV2 cells to increasing concentrations (0.1, 1, 10, or 100 ng/mL) of LPS derived from *E. coli* or *P. vulgatus*. Cell viability, assessed by MTT assay, was not significantly affected by either LPS across all tested concentrations, although at 100 ng/mL, *E. coli* LPS induced a mild but statistically significant reduction in cell viability compared to *P. vulgatus* LPS (*p* < 0.05, *E. coli* vs. *P. vulgatus*, Figure 1A). Consistently, immunofluorescence analysis of Iba-1^+^ microglial cells revealed no evident morphological alterations or significant reduction in cell number under any treatment condition, including 100 ng/mL, despite a modest trend toward reduced BV2 cell number observed following *E. coli* LPS treatment (Figures 1B,C). Notably, Iba-1 signal intensity increased in BV2 cells after *E. coli* LPS stimulation compared with the non-stimulated (NS) condition (*p* < 0.05, Figure 1D). In contrast, *P. vulgatus* LPS did not induce a comparable enhancement (Figure 1D), suggesting only a mild microglial activation profile.

Clear differences emerged even when inflammatory activation was assessed. Nitrite accumulation, used as an indirect measure of NO production, was strongly increased by *E. coli* LPS at 10 and 100 ng/mL, indicating robust NO release by BV2 cells (Figure 1E). Strikingly, this response was completely absent following stimulation with *P. vulgatus* LPS at all tested concentrations. Accordingly, *E. coli* LPS markedly increased inducible nitric oxide synthase (iNOS) expression, whereas *P. vulgatus* LPS failed to induce detectable iNOS upregulation (Figure 1F). These data indicate that *P. vulgatus* LPS neither impairs cell viability nor increases Iba1 signal intensity and, unlike canonical enterobacterial LPS, it fails to engage the NO-iNOS axis in BV2 microglia. Instead, it may promote a non-inflammatory, tolerance-like state rather than the classical activation typically associated with enterobacterial LPS (Qin et al., 2016; Chavero Vargas et al., 2025).

### *P. vulgatus* LPS fails to induce canonical cytokine production and TLR4 signaling in BV2 microglia

**FIGURE 2:**
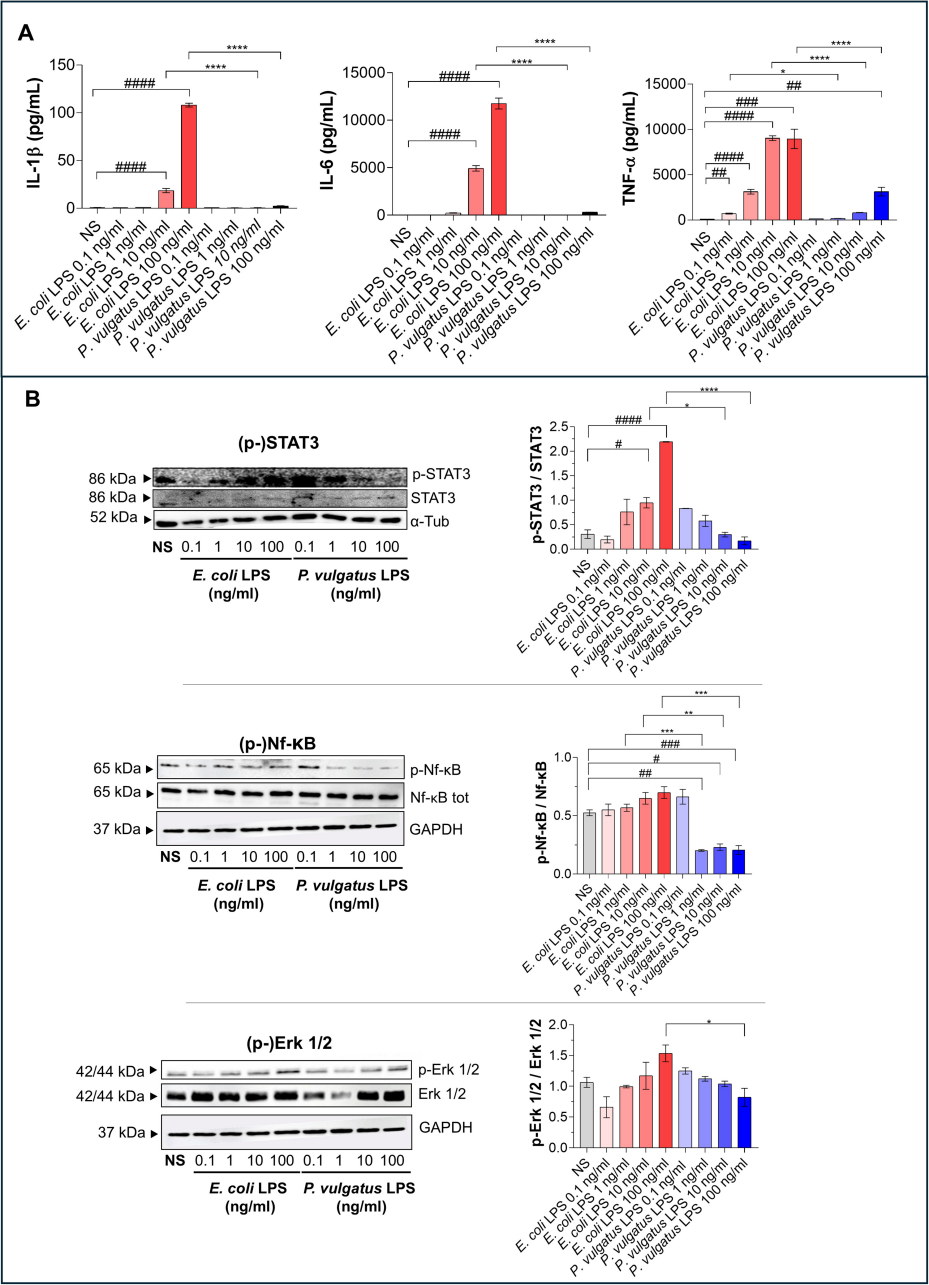
Differential modulation of cytokine release and intracellular signaling in BV2 microglia. BV2 cells were treated with increasing concentrations (0.1, 1, 10, and 100 ng/mL) of LPS derived from *E. coli* or *P. vulgatus*. Unstimulated cells (NS) served as negative controls. **(A)** Cytokine levels (IL-1β, IL-6, and TNF-α) were quantified in the culture supernatants by using ELLA assay. **(B)** Activation of intracellular signaling pathways (STAT3, Nf-kb, ERK1/2) was assessed by western blotting. Data are presented as mean ± SEM. Statistical significance was assessed by ordinary one-way ANOVA followed by Tukey’s multiple-comparison test. ^#^*p* < 0.05; ^##^*p* < 0.01; ^###^*p* < 0.001, ^####^*p* < 0.0001 vs. NS; *p < 0.05; **p < 0.01; ***p < 0.001, ****p < 0.0001 vs. LPS.

To further characterize microglial activation and to directly assess whether *P. vulgatus* LPS elicits a canonical LPS-induced inflammatory response, we measured the prototypical pro-inflammatory cytokines associated with microglia-mediated neuroinflammation (Yang et al., 2016; Colonna and Butovsky, 2017). As expected, *E. coli* LPS induced a robust, dose-dependent increase in IL-1β, IL-6, and TNF-α (Figure 2A). Conversely, *P. vulgatus* LPS induced an attenuated cytokine release profile, with only TNF-α showing only a modest increase (Figure 2A). In addition, we evaluated IL-10 release as a representative anti-inflammatory cytokine to further characterize the microglial response. However, under our experimental conditions, IL-10 levels remained very low and were often close to the detection limit of the assay (Supplementary Figure 2A).

We next examined the activation of intracellular signaling pathways downstream of TLR4 engagement. *E. coli* LPS promoted robust phosphorylation of STAT3, NF-κB, and ERK1/2 (Figure 2B), consistent with their implication in canonical LPS-induced pro-inflammatory signaling (Guha et al., 2001; He et al., 2016; Zheng et al., 2022). In contrast, *P. vulgatus* LPS resulted in markedly reduced phosphorylation of all three signaling mediators (Figure 2B). To further characterize NF-κB activation dynamics, we evaluated cytosolic p-NF-κB levels and observed a clear increase in BV2 cells following *E. coli* LPS exposure, but not after *P. vulgatus* LPS stimulation (Supplementary Figure 3). Specifically, *E. coli* LPS induced rapid phosphorylation of NF-κB as early as 5 min, at 10 and 100 ng/mL. At these early time points, p-IκB levels were low or undetectable at the highest dose (100 ng/mL), consistent with rapid IκB phosphorylation followed by degradation. By 1 h, both p-NF-κB and p-IκB signals became detectable across conditions, consistent with the well-known feedback cycle of IκB resynthesis and dynamic NF-κB signaling. Together, these findings demonstrate that *P. vulgatus* LPS fails to activate key inflammatory signaling pathways in BV2 microglia, supporting the notion that also microglia exhibit a markedly distinct response to commensal-derived versus enterobacterial LPS (Di Lorenzo et al., 2022), likely reflecting differences in TLR4 engagement or signaling dynamics.

### Human HMC3 microglia display attenuated responses to commensal-derived LPS compared to enterobacterial LPS

**FIGURE 3:**
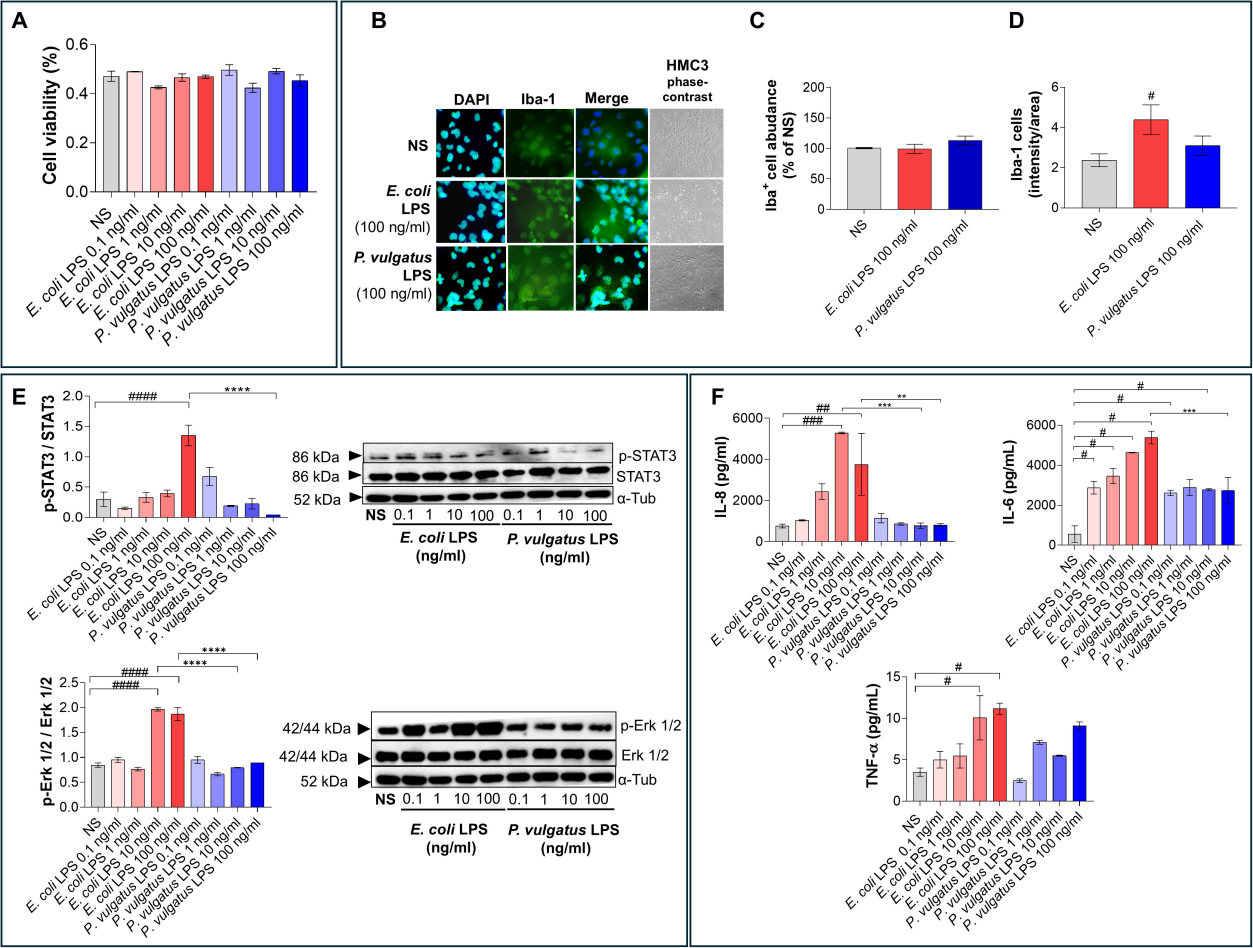
Differential responses of human HMC3 microglia to LPS from *E. coli* and *P. vulgatus*. **(A)** HMC3 cell viability was assessed after stimulation with increasing concentrations of LPS (0.1, 1, 10, and 100 ng/mL) from *E. coli* or *P. vulgatus*, using the MTT assay. Unstimulated cells (NS) were used as negative control. **(B)** Representative immunofluorescence Iba-1^+^ cells (green) and corresponding brightfield phase-contrast microscopy images of HMC3 cells (scale bar = 100 μm). **(C)** Quantification of Iba-1^+^ cell number from the experiments in B, expressed as percentage relative to NS condition (set at 100%). **(D)** Iba-1 immunofluorescence intensity calculated as the mean area of the positive signal per cell. **(E)** Western blot analysis of TLR4-associated signaling pathways. **(F)** Cytokine levels (IL-8, IL-6, TNF-α) measured in culture supernatants by ELLA assay. Data are presented as mean ± SEM. Statistical significance was assessed by ordinary one-way ANOVA followed by Tukey’s multiple-comparison test. ^#^*p* < 0.05; ^##^*p* < 0.01; ^###^*p* < 0.001, ^####^*p* < 0.0001 vs. NS; **p < 0.01; ***p < 0.001, ****p < 0.0001 vs. LPS.

To validate the observations obtained in murine microglia, we next tested the effects of *E. coli* and *P. vulgatus* LPS on HMC3 cells, a human microglial cell line known to exhibit limited classical inflammatory responses and commonly used to model human-specific microglial behavior (Janabi et al., 1995; Dello Russo et al., 2018). Across the tested concentration range, neither LPS significantly affected HMC3 cell viability (Figure 3A) nor induced overt morphological alterations (Figure 3B). No statistically significant differences in total Iba-1^+^ cell number were detected across treatments, although a modest trend toward an increased number of Iba-1^+^ cells was observed in *P. vulgatus* LPS-treated cultures (Figure 3C). Similar to BV2 cells, HMC3 cells displayed increased Iba-1 signal intensity following *E. coli* LPS stimulation compared with the NS condition (Figure 3D). Conversely, *P. vulgatus* LPS did not produce a comparable increase in this cell line either, further supporting a markedly milder microglial activation profile.

Nitrite production was not detected in HMC3 cells under any experimental conditions, in agreement with previous studies showing that HMC3 cells lack iNOS induction and do not produce NO even upon classical pro-inflammatory stimulation (Li et al., 2009; Hjorth et al., 2010; Landry et al., 2012; Jadhav et al., 2014; Dello Russo et al., 2018; De Chirico et al., 2022). Accordingly, microglial activation was assessed using alternative and more informative inflammatory readouts. Western blot analysis revealed that *P. vulgatus* LPS induced markedly lower phosphorylation of STAT3 and ERK1/2 compared to *E. coli* LPS (Figure 3E), indicating a strongly attenuated engagement of TLR4-associated signaling pathways also in this cell line. NF-κB activation could not be reliably assessed in HMC3 cells under our experimental conditions (data not shown), consistent with known limitations of this cell line in activating NF-κB-dependent transcriptional programs (Dello Russo et al., 2018; De Chirico et al., 2022).

In parallel, as for BV2 cells, we quantified the release of pro-inflammatory cytokines typically analyzed in LPS-induced neuroinflammation models. In HMC3 cells, we focused on IL-8, IL-6, and TNF-α, selecting IL-8 in place of IL-1β because IL-8 is known to be more robustly and consistently produced by this cell line, whereas IL-1β expression is typically low or undetectable (Dello Russo et al., 2018). Intriguingly and consistent with the signaling data, *P. vulgatus* LPS elicited significantly lower cytokine release compared with *E. coli* LPS (Figure 3F). Finally, similarly to BV2 cells, HMC3 cells also display negligible IL-10 secretion (Supplementary Figure 2B), as previously reported for this cell line (Dello Russo et al., 2018). Collectively, these results demonstrate that, even in a human microglial model with limited classical inflammatory responsiveness, microglia can discriminate between structurally distinct LPS chemotypes, and that *P. vulgatus* LPS fails to induce a canonical pro-inflammatory microglial program.

### Effect of LPS-treated HMC3 microglial supernatant on PC12 neuronal cells

Beyond their defensive role, microglia play a critical role in neuroprotection, establishing and maintaining brain homeostasis (Graeber et al., 2011; Ransohoff et al., 2015). However, chronic activation of microglia becomes detrimental, contributing to neuronal dysfunction in neuroinflammatory settings (Neumann et al., 2009). This is largely due to the sustained release of cytotoxic mediators, including pro-inflammatory cytokines and reactive oxygen or nitrogen species (Block et al., 2007; Hughes, 2012; Dai et al., 2015; Gao et al., 2023), which have been implicated in the progression of several neurodegenerative diseases (Hughes, 2012). Although the mechanisms underlying microglia-neuron communication are not yet fully understood, TLR4-dependent microglial activation by LPS is a well-established driver of neuronal injury across multiple *in vivo* models (Lehnardt et al., 2008; Singh et al., 2022; Skrzypczak-Wiercioch and Sałat, 2022).

**FIGURE 4:**
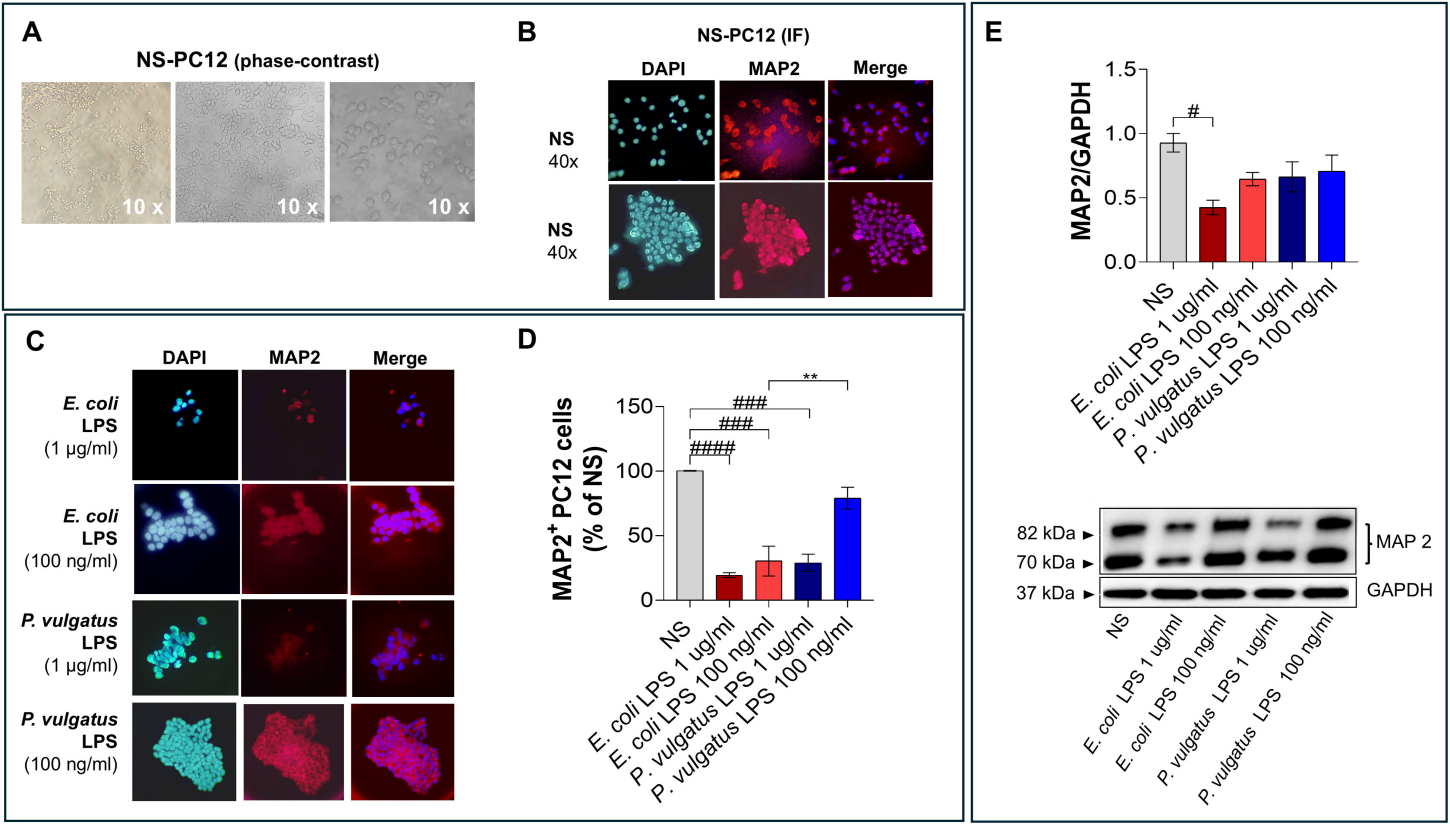
Effects of LPS-treated HMC3 conditioned media on neuronal integrity **(A)** Bright-field images of differentiated PC12 cells (10 × and 40 × magnification) and **(B)** MAP2 immunofluorescence staining. **(C)** Immunofluorescence analysis was assessed after exposure to conditioned media (CM) from HMC3 cells treated with 1 μg/mL or 100 ng/mL. LPS from *E. coli* or *P. vulgatus*. Unstimulated PC12 cells (NS) from the experiments in **(B)** served as control and **(D)** the accompanying quantification confirms the differences observed. **(E)** Western blot analysis of MAP2 protein levels in PC12 cells exposed to CM from HMC3 cells treated with 1 μg/mL LPS. Data are presented as mean ± SEM. Statistical significance was assessed by ordinary one-way ANOVA followed by Tukey’s multiple-comparison test. ^#^*p* < 0.05; ^###^*p* < 0.001, ^####^*p* < 0.0001 vs. NS; **p < 0.01; vs. LPS.

Given the attenuated microglial response elicited by *P. vulgatus* LPS, we next investigated whether this differential activation profile translated into distinct downstream effects on neuronal cells. HMC3 cells were therefore stimulated with *E. coli* or *P. vulgatus* LPS (100 ng/mL or 1 μg/mL), and the resulting supernatants were collected and used as conditioned media (CM) to treat differentiated PC12 neuronal cells, a useful model system for neurobiological and neurochemical studies (Greene and Tischler, 1976). Concentrations were selected based on preliminary dose-response experiments, which identified 100 ng/mL as the most effective dose in eliciting cellular responses. In addition, 1 μg/mL LPS was included as it represents a widely used *in vitro* pro-inflammatory stimulus to ensure robust microglial activation and the generation of conditioned media capable of eliciting detectable neuronal responses, as commonly reported in studies of microglia-neuron interactions (Lajqi et al., 2019; He et al., 2021). PC12 cells were differentiated using NGF and after brightfield phase-contrast morphological assessment (Figure 4A), neuronal differentiation was confirmed via immunofluorescence staining for MAP2 (Figure 4B; González-Billault et al., 2002; Dehmelt and Halpain, 2005). Despite PC12 cells exhibited a tendency to organize into cellular clusters with limited neurite outgrowth (Figure 4A), MAP2 immunoreactivity observed in these cells is consistent with successful neuronal differentiation (Figure 4B).

Cell viability was also assessed by MTT assay, showing that neither LPS significantly affected cell viability (Supplementary Figure 4). Immunofluorescence analysis also revealed that exposure to CM derived from *E. coli* LPS-treated HMC3 cells significantly reduced the number of MAP2^+^ PC12 cells, even at 100 ng/mL, suggesting impaired neuronal differentiation or maintenance (Figures 4C,D). In contrast, CM from *P. vulgatus* LPS-treated microglia had no significant effect on MAP2 expression at the same concentration, when compared to both unstimulated controls and the *E. coli* LPS condition (Figures 4C,D). These findings were further supported by western blot analysis, which confirmed a marked decrease in MAP2 protein levels in PC12 cells exposed to *E. coli*-derived CM (1 μg/mL), while only a modest reduction was observed following treatment with CM from *P. vulgatus* LPS-stimulated microglia (Figure 4E). While minor differences between the two readouts likely reflect methodological sensitivity and the qualitative versus quantitative nature of immunofluorescence and immunoblotting, respectively (Vilibic-Cavlek et al., 2021), both approaches converge on the same biological conclusion: microglial factors released in response to *E. coli* LPS impair neuronal integrity, whereas those released following *P. vulgatus* LPS exposure do not.

### *P. vulgatus* LPS is integrated into systemic immune recognition in the absence of inflammation

To place our microglial findings within a broader human immunological context, we next examined whether *P. vulgatus* LPS represents a microbial signal that is physiologically recognized by the host immune system under homeostatic conditions. Using an LPS-coated ELISA (Gleńska-Olender et al., 2017), we detected circulating anti-LPS IgG antibodies in plasma samples from healthy donors (a schematic representation of experimental procedure is presented in Supplementary Figure 5), evaluating prior immune recognition of gut-derived LPS in individuals without explicit inflammatory disease. Notably, IgG reactivity against *P. vulgatus* LPS was significantly higher than that observed against *E. coli* LPS or non-coated controls (Supplementary Figure 5). This antibody profile is consistent with sustained, non-inflammatory immune recognition, potentially reflecting immune tolerance or regulatory engagement with a commensal-derived LPS chemotype. In the context of microglial biology, this distinction is relevant, as it suggests that *P. vulgatus* LPS corresponds to a molecular pattern that is integrated into the host baseline immunological milieu rather than acting as a classical danger signal. Combined with our *in vitro* findings, these results support the view that *P. vulgatus* LPS belongs to a distinct class of LPS chemotypes compatible with immune homeostasis. This immune context provides an essential background for interpreting the attenuated microglial activation and lack of neurotoxic effects observed in response to *P. vulgatus* LPS, further reinforcing the notion that microglial inflammatory outcomes are not a generic reaction to any LPS, but a selective response shaped by the molecule precise chemical architecture.

## Discussion

The present study demonstrates that microglia do not respond indiscriminately to LPSs, but instead interpret LPS chemistry, translating subtle structural differences into sharply divergent functional outcomes. While canonical enterobacterial LPS, such as that from *E. coli*, triggered strong activation of inflammatory pathways and cytokine production, *P. vulgatus* LPS failed to elicit a comparable response in both murine and human microglial models. This attenuated activity is consistent with the peculiar chemical architecture of this LPS. In particular, its hypoacylated and monophosphorylated lipid A structure is likely a major determinant of its reduced capacity to activate TLR4-dependent signaling (Mercogliano et al., 2026). In addition, the unusual glycan portion of this LPS may engage carbohydrate-recognizing immune receptors, including C-type lectins such as DC-SIGN, which have been reported to modulate immune responses (Nieto-Fabregat et al., 2024; Rai et al., 2025). Unlike TLR4, DC-SIGN does not directly trigger NF-κB activation but can modulate TLR-driven signaling pathways and promote regulatory programs, including IL-10 production (Nieto-Fabregat et al., 2024; Rai et al., 2025). Furthermore, previous studies suggest that this LPS may also engage TLR2 and TLR4 in a cooperative manner (Di Lorenzo et al., 2020), potentially adding an additional layer of modulation beyond the canonical inflammatory signaling typically induced by enterobacterial LPS. Taken together, these observations, along with the results presented in this study, challenge the widespread assumption that all LPS molecules act as inherently pro-inflammatory stimuli in neuroinflammation research.

Our data clearly demonstrate that *P. vulgatus* LPS does not induce NO production, iNOS expression, or a robust release of classical cytokines (IL-1β, IL-6, TNF-α) in BV2 microglia. Consistent with its structural features, *P. vulgatus* LPS also fails to activate key TLR4-associated signaling pathways, including STAT3 and ERK1/2. Notably, this attenuated profile was not limited to rodent microglia. Human HMC3 cells, despite their well-documented limitations in classical TLR4 signaling (Janabi et al., 1995; Dello Russo et al., 2018; De Chirico et al., 2022), also discriminated between the two LPS chemotypes. This observation suggests that other receptors beyond TLR4 may be involved in sensing *P. vulgatus* LPS, consistent with prior reports on this structurally distinct molecule (Steimle et al., 2019; Di Lorenzo et al., 2020; Nieto-Fabregat et al., 2024; Rai et al., 2025; Mercogliano et al., 2026). Moreover, *E. coli* LPS stimulation significantly increased the Iba-1 signal intensity in both BV2 and HMC3 cells, indicating a strong microglial activation response. In contrast, *P. vulgatus* LPS did not induce a comparable increase in Iba-1 intensity, suggesting it triggers only mild microglial activation. Overall, these findings suggest that the divergent microglial responses observed are not model-specific but reflect a broader principle of structure-dependent immune recognition.

These differences in microglial sensing were not without consequence: they translated into strikingly different effects on neuronal health. Conditioned media from *E. coli* LPS-treated microglia significantly disrupted MAP2 expression in differentiated PC12 neurons. By contrast, *P. vulgatus* LPS-derived media exerted a milder and more attenuated effect, with MAP2 levels largely preserved. These results provide functional evidence that only specific LPS chemotypes propagate neurotoxic cascades via microglial activation, underscoring the need for structure-aware modeling in neuroinflammatory contexts. This also aligns with and expands upon findings by Vargas-Caraveo et al. (2020), who showed that *E. coli* LPS strongly activates the inflammatory response in circumventricular organs of rats, promoting NF-κB nuclear translocation and inducing a shift in microglial morphology toward an ameboid, reactive state. In contrast, LPS from *P. gingivalis*, a weaker TLR4 agonist, triggered NF-κB translocation only in the median eminence, while the TLR4 antagonist LPS from *R. sphaeroides* failed to activate NF-κB in any CVO but unexpectedly promoted a rod-like microglial morphology, typically associated with severe neuropathology. These prior observations already pointed to the idea that microglial responses to LPS are tightly linked to structural features of the molecule, a dimension still largely underexplored in the context of brain immunity.

Given the growing recognition that many neuroinflammatory disorders are increasingly associated with alterations in gut microbiota composition (Ma et al., 2019; Suganya and Koo, 2020; Morais et al., 2021; Loh et al., 2024; Keane et al., 2025), the gut-brain axis has emerged as a critical framework for understanding how microbial signals influence CNS function. In this frame, where host exposure is dominated by structurally diverse gut-derived commensal LPSs rather than pathogenic ones, reliance on highly immunostimulatory enterobacterial chemotypes may profoundly distort biological interpretation. As a matter of fact, the detection of circulating anti-*P. vulgatus* LPS IgG in healthy individuals suggests regular, non-pathological exposure to this commensal-derived LPS, and its integration into the host immunological landscape. Within this context, microglia appear capable of sensing LPSs from specific commensals without engaging inflammatory or neurotoxic pathways, reinforcing the concept that microbial glycans can actively shape, rather than disrupt, neural immune tone.

Some limitations should be acknowledged. First, NF-κB activation was quantified only in the cytosolic fraction of BV2 cells, and nuclear NF-κB translocation, which would provide a more complete assessment of pathway activation, was not evaluated. Moreover, the use of immortalized microglial cell lines, while advantageous for controlled comparative and mechanistic analyses, may not fully capture the diversity and functional complexity of primary microglia *in vivo*. Compared with primary microglia, these cell lines can exhibit altered basal activation states, proliferative behavior, and cytokine expression profiles, and may differ in TLR expression and signaling sensitivity. However, future studies integrating flow cytometry analyses with established microglial activation markers (e.g., CD38, CD40, CD206) could provide a more detailed characterization of the phenotypic shifts induced by distinct LPS chemotypes. Likewise, although PC12 cells represent a widely used neuronal differentiation model, they may not encompass the full spectrum of neuronal phenotypes relevant to neuroinflammation. While NGF-differentiated PC12 cells express several neuronal markers, a broader phenotypic characterization could provide additional insight. Nevertheless, the consistency of our findings across different microglial models and experimental approaches provides a coherent framework supporting the robustness of the observed differential responses to distinct LPS chemotypes.

Together, these findings carry several important implications. First, they argue against the continued reliance on highly immunostimulatory *E. coli* LPS as a generic tool for modeling microglial activation. Such models, though experimentally convenient, may distort biological relevance especially in studies probing gut-derived contributions to CNS inflammation. Second, our results highlight the need for a more refined understanding of “LPS-induced neuroinflammation” as a context- and chemistry-dependent process, rather than a generic outcome. Finally, they support a growing body of evidence suggesting that microbial glycans, including structurally distinct forms of LPS, can act as regulatory signals within the brain immune environment.

Recognizing and incorporating LPS structural diversity is therefore essential, not only for interpreting microglial behavior accurately, but also for developing more realistic models of neuroinflammation and understanding how microbial signals from the gut shape brain health and disease.

## Footnotes

1
https://imagej.nih.gov/ij/
