# Supplementary material for: Structure matters: commensal Phocaeicola vulgatus lipopolysaccharide induces attenuated microglial activation and preserves neuronal integrity
Source: Front Cell Neurosci. 2026 Apr 14;20:1796397. doi: 10.3389/fncel.2026.1796397 (PMC13120944; doi:10.3389/fncel.2026.1796397)
Supplement: Supplementary file 1 [file Data_Sheet_1.pdf]

Supplementary Material

Structure matters: commensal *Phocaeicola vulgatus* lipopolysaccharide induces attenuated microglial activation and preserves neuronal integrity

Valentina Mazziotti, Luca De Simone Carone, Francesca Olmeo, Fabrizio Chiodo, Alba Silipo, Antonio Molinaro, Flaviana Di Lorenzo

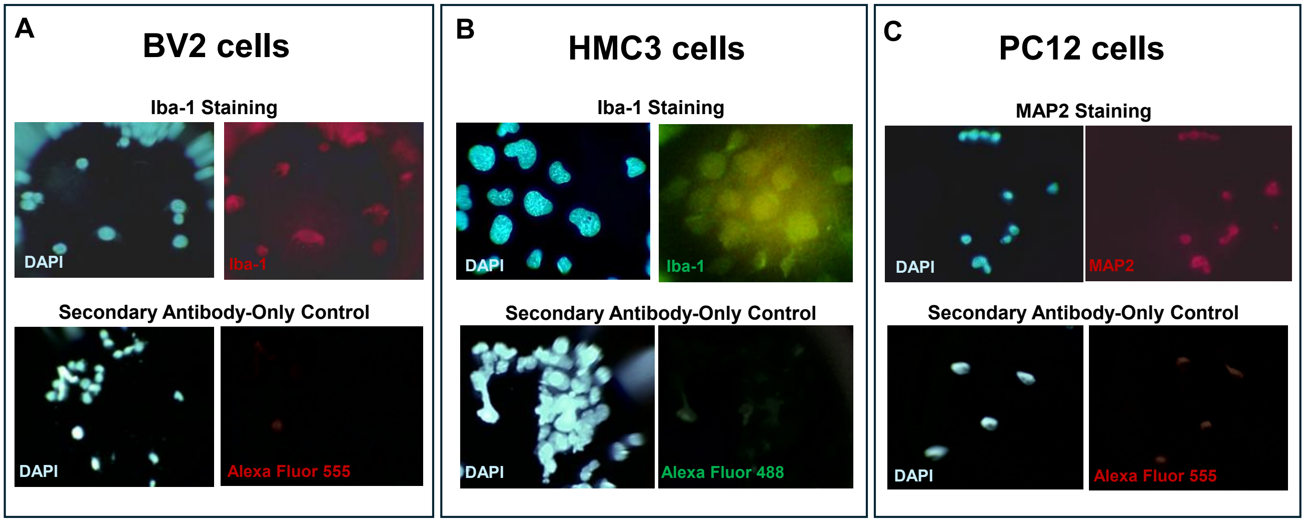

Supplementary Figure S1. Secondary antibody-only control for immunofluorescence. Representative images of Iba-1 staining in BV2 (**A**) and HMC3 (**B**) microglial cells, and MAP2 staining in PC12 (**C**) neuronal cells, are shown below their corresponding secondary-only controls. For negative controls, samples were processed identically to experimental conditions but incubated exclusively with the fluorescent secondary antibody, in the absence of primary antibodies.

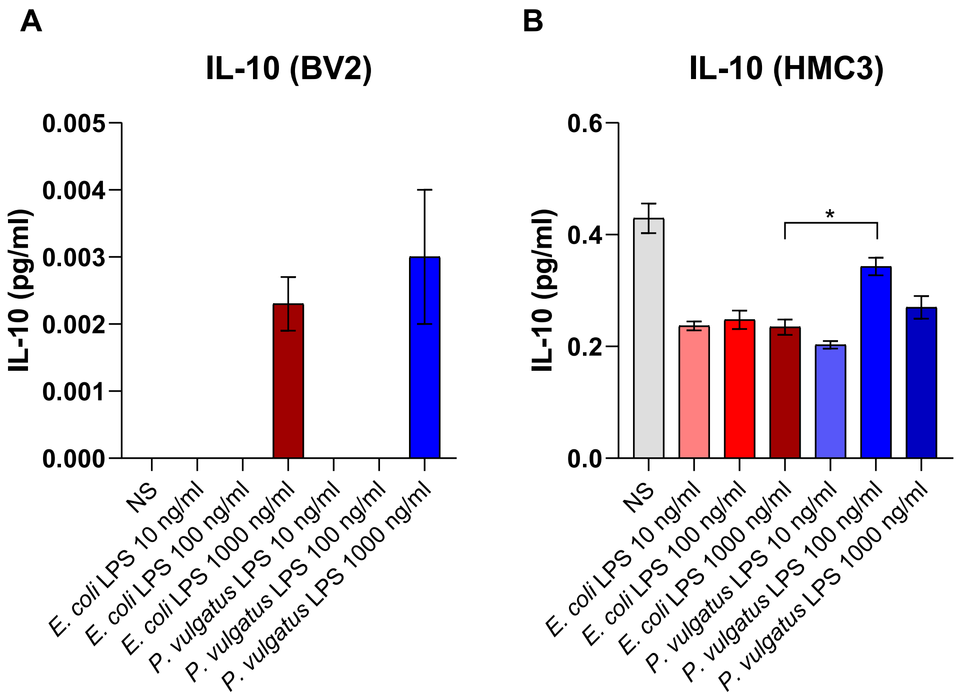

Supplementary Figure S2. IL-10 release by microglial cells. IL-10 quantified in the supernatants of BV2 (**A**) and HMC3 (**B**) cell cultures by using DuoSet ELISA Kit. **p* < 0.05 vs LPS.

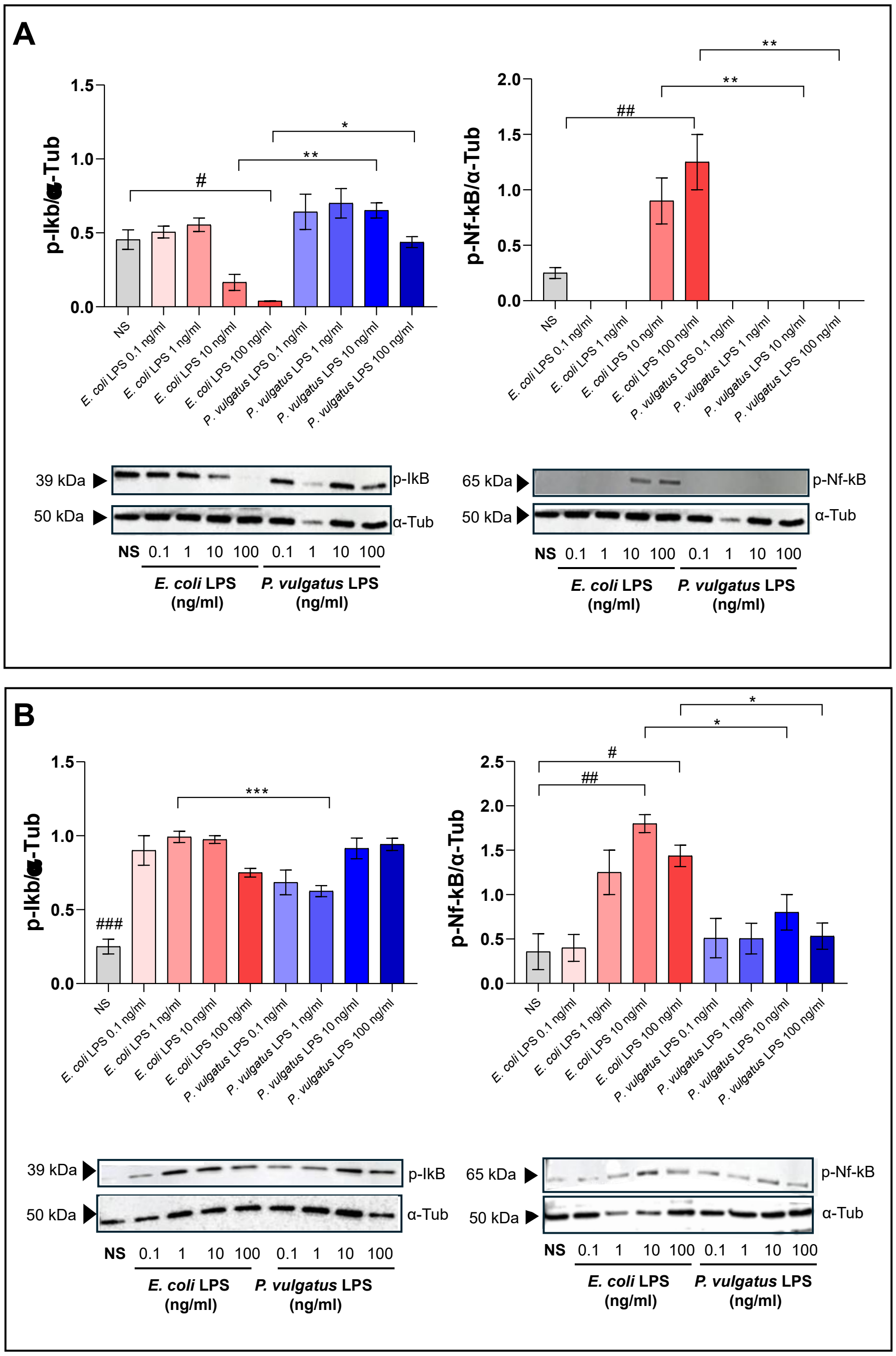

Supplementary Figure S3. Effects of *E. coli* and *P. vulgatus* LPS on p-NF-κB and p-IκB protein levels in BV2 microglia. Level of p-IκB and p-NF-κB in BV2 cells after 5 min (**A**) and 1 hour (**B**) of exposure to 0.1, 1, 10 and 100 ng/ml LPS by Western blotting. $^{\#}p < 0.05$; $^{\#\#}p < 0.01$ vs NS; $^{*}p < 0.05$; $^{**}p < 0.01$; $^{***}p < 0.001$, vs LPS

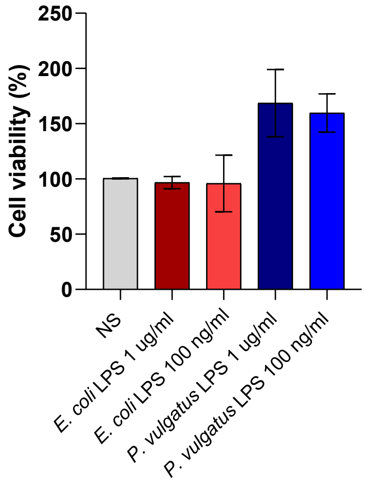

Supplementary Figure S4. Effects of *E. coli* and *P. vulgatus* LPS on PC12 cell viability. Cell viability was assessed after stimulation with increasing concentrations of *E. coli* or *P. vulgatus* LPS (100 ng and 1 ug) using the MTT assay.

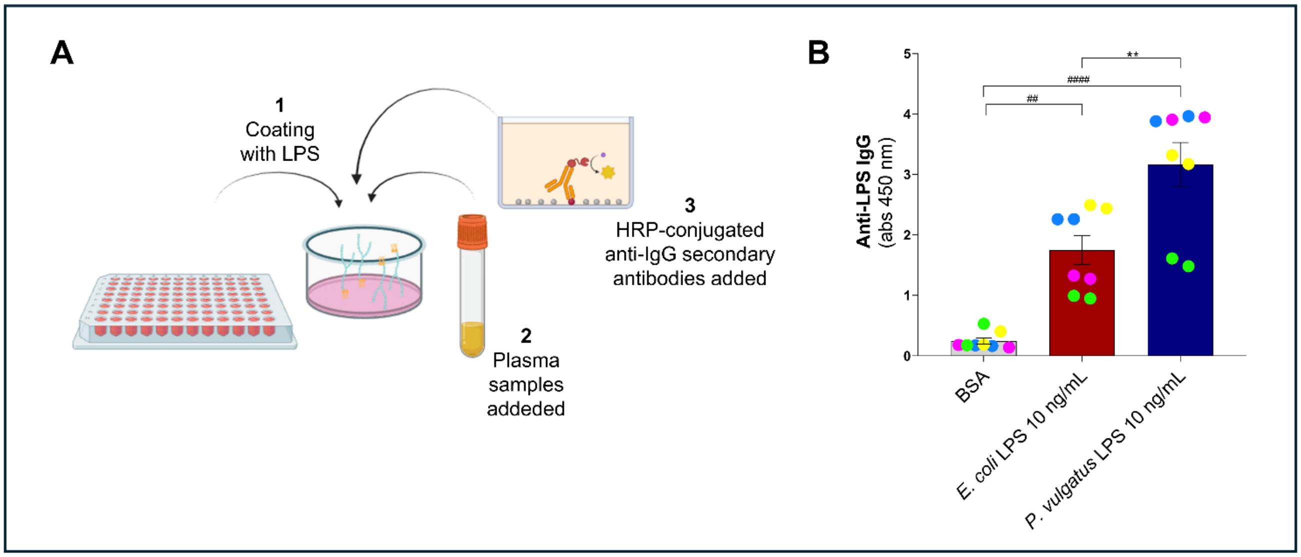

Supplementary Figure S5. Evidence of systemic exposure to *P. vulgatus* LPS in healthy donors. (**A**) Schematic representation of the ELISA assay used to detect anti-LPS IgG antibodies in human plasma. Plates were coated with 10 ug/mL of purified LPS from *E. coli* or *P. vulgatus*. This illustration was created using BioRender. (**B**) Quantification of anti-LPS IgG levels after LPS treated measured by LPS-coated ELISA assay: increased anti-*P. vulgatus* LPS IgG were detected in plasma samples from n=4 healthy donors compared with NS and *E. coli* LPS conditions data are presented as mean ± SEM. Statistical significance was assessed by ordinary one-way ANOVA followed by Tukey's multiple-comparison test.$^{##}p < 0.01$; $^{###}p < 0.001$ vs NS; $^{**}p < 0.01$vs LPS
